# Water mediated dielectric polarizability and electron charge transport properties of high resistance natural fibers

**DOI:** 10.1038/s41598-018-20313-4

**Published:** 2018-02-09

**Authors:** Ankit Kumar, Amit Jash, Amarish Dubey, Alok Bajpai, Deepu Philip, Kalpana Bhargava, Sushil K. Singh, Mainak Das, S. S. Banerjee

**Affiliations:** 1 Department of Physics, Indian Institute of Technology, Kanpur, 208016 Uttar Pradesh India; 2 Design Program, Indian Institute of Technology, Kanpur, 208016 Uttar Pradesh India; 3 Psychiatrist, Medical Centre, Indian Institute of Technology Kanpur, Kanpur, 208016 Uttar Pradesh India; 4 Industrial and Management Engineering, Indian Institute of Technology Kanpur, Kanpur, 208016 Uttar Pradesh India; 5 DRDO, Timarpur, Delhi, 110054 India; 6 Biological Sciences & Bioengineering, Indian Institute of Technology Kanpur, Kanpur, 208016 Uttar Pradesh India

## Abstract

Recent studies showed that silk and human hair fibers develop thermoelectric properties at optimal water, temperature and light conditions. The nature of charge carriers and the role of water in mediating charge conduction in these fibers is an unexplored issue. By studying four different classes of natural fibers, viz., silk cocoon, human hair, jute and corn silk, we uncover their common electrical transport properties and its dependence on water concentration and temperature. All these fibers uniformly exhibit nonlinear, hysteretic current - voltage characteristics, which scale with water concentration. The optimal electrical conductivity shows thermally activated hopping transport mechanism. Scanning tunneling microscope (STM) and dielectric measurements of silk cocoon fibers showed the electronic density of states and dielectric properties of the hydrated medium enhances with water concentration. Electron paramagnetic resonance (EPR) study reveals that the charge carriers in these membranes are electronic in nature. Our results are explained through the mechanism of hopping of a Polaron, which is an electron surrounded by positive charge fluctuations created by water molecules. The mechanism unravels the peculiar role water plays in mediating electrical activity in these membranes and also opens the possibility for exploring such charge transport mechanism in other biological membranes.

## Introduction

Recent studies show that silk and human hair fibers have novel thermoelectric properties^1,2^. Understanding the electrical properties of hydrated biological membranes is important not only for applications but also for its fundamental relevance in investigating the behavior of electrical circuitry in the natural living world. In biological membranes charge carriers can be either electrons or protons^3^ and charge conduction occurs in an aqueous medium. One may ask if water is necessary for charge transport in such systems? It is well known that water is a vital ingredient in sustaining biological and chemical functions in plant and animal tissues^4–6^. Water is important to maintain protein functionality^7^, regulating their shape^8^ and mobility^9,10^. Water also affects protonic charge transport across proteins. Studies suggest that the transport of protons across biological macromolecules^4,7,11^ like proteins occur via the formation of an intermediate ionic complex of water molecules (e.g. (H_5_O_2_)^+^ species)^11–21^. The charges are considered to hop along proton wires created on the hydrated molecular backbone of biological macromolecules. While the role of water, in mediating proton charge transport has been widely investigated, its role in promoting electron charge transport in such systems has not been well explored.

The recent experiments on the most abundant natural fibers from insect, animal and plant kingdom^22–26^ viz., silk cocoon (*Bombyx mori*, *Antheraea mylitta*), human hair, jute and corn-silk (obtained from the corn cob of the maize plant) show that at optimum moisture, temperature and light conditions, these fibers exhibit thermoelectric, photo-electric and magnetic properties^1,2,27,28^. However, the nature of charge carriers and the effect of water (if any) on the charge transport properties of these fibers, have not been explored. To address the above questions, we study the electrical properties of four natural fibers, viz., silk cocoon, human hair, jute and corn-silk. These fibers represent four distinct class of chemical families of fibers^22–26^, viz., silk cocoon is a beta-sheet fibroin protein forming a composite structure by adhering with a glue like protein viz., sericin^22,23^, human hair represents alpha-helical protein^24^, jute represents a pure carbohydrate chain^25^ and corn-silk represents carbohydrate and protein hybrid^26^ (see Methods and also the section: Image of samples with electrical contacts, in Supplementary Information). In our measurements, the fibers are maintained in their native state without denaturing them in any way. All membranes exhibit low electrical conductivity, which improves with increasing water content. The membranes have a nonlinear, hysteretic current (*I*) - voltage (*V*) characteristics, where the hysteresis scales with the water content. Temperature dependent electrical conductivity reveals a thermally activated charge transport mechanism in these natural membranes. Our scanning tunneling microscope (STM) measurements show strong water induced enhancement in the electronic density of states and the development of a local internal electric field within the membrane. We show these highly resistive membranes develop significant dielectric polarizability with a large dielectric constant ~169 in the presence of water. The dielectric constant decreases with reducing water content in these hydrated membranes. Electron paramagnetic resonance (EPR) measurements unravel the electronic nature of the charge carriers in these membranes and also the presence of water weakens the coupling of the charges with their complex chemical environment. We model the charge transport in these hydrated dielectric membranes occurs via the model of hopping of a Polaron complex.

## Results

### Bulk electrical characterization of the biological membranes using transport measurement

**Figure 1:**
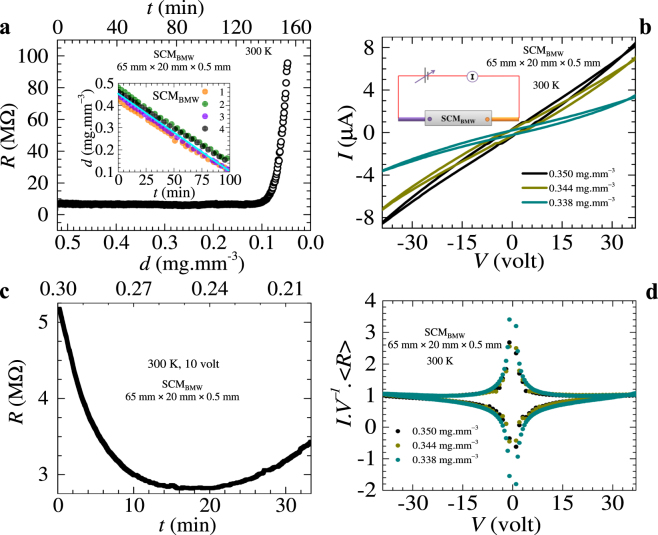
Electrical transport study of hydrated SCM_BMW_. For electrical transport measurement, we have used SCM_BMW_ (consisting of natural fibers obtained from the *Bombyx mori* silk cocoon). The membrane has dimensions of 65 mm (length) × 20 mm (width) × 0.5 mm (thickness). For all our measurements, electrical contacts are made directly on the membrane at its two ends. The distance between the contacts is approximately 61 mm in present case. Since the resistance of these fibers is very high, the electrical transport measurements are of two probe type. All electrical measurements are done at room temperature. In all our figures, the error bars are smaller than the size of the data symbols. (**a**) Shows the dependence of resistance (*R*) (left axis) of hydrated SCM_BMW_ on the water concentration (*d*) (bottom axis) which varies with time (*t*) (top axis). Notably, there is a rapid change in the resistance as the water concentration in SCM_BMW_ falls below a critical value *d*_*c*_ = 0.1 mg.mm^−3^. The inset of (**a**) shows four different runs, 1 (orange), 2 (olive), 3 (violet) and 4 (black) depicting the linear behavior of water concentration as a function of time in SCM_BMW_ with a slope (cyan color) of m = − (5.28 ± 0.04) × 10^−5^ mg.mm^−3^.sec^−1^. (**b**) The inset is a schematic of the two probe electrical contact on hydrated SCM_BMW_. The main panel shows a hysteresis in the *IV* characteristics of the SCM_BMW_ measured with different water concentration (the *d* values are shown). (**c**) Shows the resistance (*R*) measured as a function of time (see bottom axis) or, by using the linear *d*(*t*) relation, as a function of *d* (top axis). Here *R* is measured with 10 volts applied across the contacts on the SCM_BMW_. In Fig. 1c we begin the *R*(*t*) measurement with a fresh SCM_BMW_ with an initial water concentration of 0.3 mg.mm^−3^ and then it is allowed to dry naturally. For clarity in seeing the *R*(*d*) behavior, the measurement is performed over a relatively shorter range of variation in *d* (compared to that in Fig. 1a). We see that *R* is sensitive to the membrane water concentration and seems to show minima at intermediate water concentration. (**d**) The *IV* data in Fig. 1b is normalized and replotted as *I*.*V*^−1^. <*R*> vs. *V*. The figure shows that all the different *IV* curves of (**b**) scale onto a single master curve. The scaling property of these curves implies that the *IV* hysteresis changes monotonically with water concentrations (*d*) of the membrane and the shape of the hysteresis also remains unchanged at different values of *d*.

Here we study the bulk electrical conducting properties of natural membranes made from a collection of *Bombyx mori* silk cocoon (SCM_BMW_) fibers (obtained from the silk cocoon). The electrical properties of these fibers are measured by making traditional electrical contacts directly on the membrane (see Methods). The fibers are maintained in their native state and all the measurements are performed under controlled hydration levels (*d*). The dry silk cocoon membrane is an insulating material with a resistance *R* > 50 MΩ. Figure 1a shows that when the water content in the membrane is above a critical value *d*_*c*_ ~0.1 mg.mm^−3^, the resistance (*R*) appears to be almost uniform. Note that below *d*_*c*_ the membrane resistance diverges rapidly (Fig. 1a).

The inset of Fig. 1a shows that the water content (*d*) of the membrane depletes linearly with time (*t*). The value of *d* at *t* = 0, corresponds to the situation when the membrane is saturated with water. For a time interval of 100 minutes from the start of the experiment when the membrane is dipped in water and lifted, the wet silk cocoon membrane exhibits a linear *d*(*t*) relationship. However, at small *d* values the *d*(*t*) asymptotically approaches zero with a nonlinear tail like feature (see Extended Data Fig. 1). Note all our measurements have been performed within the time interval where *d*(*t*) behavior is linear. Although the resistance of the hydrated membrane is high, for an applied voltage of 10 V, a small current (*I*) ~10 µA passes across the membrane. The observation that *R* changes with *d* motivated our study of *IV* characteristics of these membranes at different hydration levels (*d*) (see Methods for electrical measurement). The *IV* curves for different *d* in Fig. 1b are non-linear. The shape of the nonlinear *IV* curves are similar to those studied in molecular junctions^29,30^. A noteworthy feature in Fig. 1b, is the hysteresis in *IV’s*, viz., slightly different *IV* paths are traced while changing *V* (d.c.) from −40 V to +40 V followed by +40 V to −40 V. In Fig. 1b we observe that the mean slope (which is the inverse of the average membrane resistance <*R*>^−1^) of the *IV* curve reduces with decreasing *d*, suggesting that the average membrane resistance increases with decreasing water concentration. In Fig. 1a as the variation of *R* spans a wide range, hence it appears to be almost constant at ~5 MΩ for variations in *d* > *d*_*c*_. In Fig. 1c we show the *R*(*d*) measured over a narrower range of *d* values, which is akin to viewing the *R*(*d*) behavior in Fig. 1a, on an expanded scale. Here we see the electrical resistance (*R*) is sensitive to relatively small changes in the membrane’s water concentration, in fact, *R*(*d*) becomes minimum at intermediate values of *d*. The hysteresis in Fig. 1b is explored further in Fig. 1d by replotting the *IV* data in Fig. 1b as a dimensionless variable, *I.V*^−*1*^. <*R*> versus *V* (see Methods for scaling analysis details). We found that all the hysteretic *IV* curves in Fig. 1b at different water concentrations collapsed onto a single master curve which is independent of the variation in *d*. We have shown that the value of resistance of the membrane is sensitive to variation in *d*, therefore normalizing the *IV* data of Fig. 1b using <*R*> value results in the implicit dependence on *d* being removed and hence all the curves get scaled. The scaling suggests that the shape of hysteresis is unchanged with *d* variation, which means the mechanism of charge transport remains unmodified as the water concentration of the membrane changes. The hysteresis in *IV* also indicates that electric charges flow along trajectories with different resistance as the voltage applied across the membrane is increased or decreased. Due to the complexity of these biological systems, one may expect that their properties are subjected to large variability. Hence, one may ask which aspects of Fig. 1 represents transport properties common to hydrated natural membranes? To answer this question, in the Extended Data Fig. 2 we show the hysteretic nature of the *IV* curves measured in different samples of hydrated SCM_BMW_.

**Figure 2:**
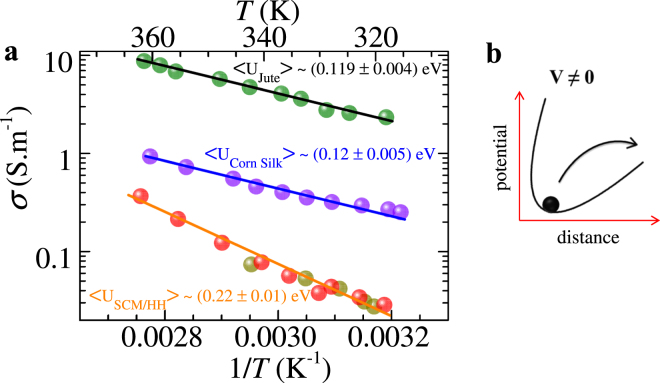
Temperature dependence of conductance. (**a**) Behavior of the log of electrical conductivity (*σ*) versus inverse of temperature (*T*) (the corresponding original temperature is shown as the top x-axis of the plot for convenience) for SCM_BMW_ (dark yellow, length = 33 mm, width = 14 mm, thickness = 0.5 mm), human hair (red, length = 40 mm, bundle diameter = 0.5 mm), corn-silk (violet, length = 61 mm, bundle diameter = 0.5 mm) and jute (olive, length = 59 mm, bundle diameter = 0.5 mm). In all these measurements, contacts are made at the two ends of the samples (distance between contacts ≈ length of the samples). Here we have chosen the symbol size comparable to the size of the error bars. For these measurements, note the value of *d* of the membranes at different *T* were maintained uniform during our entire measurement of *σ* vs *T*. This figure shows the electrical conductivity, *σ*(*T*), of the hydrated fibers obeys the Arrhenius law, $\sigma \propto \exp (- < U > /{k}_{B}T)$. The solid lines represent a linear fit to the data on the log *σ* vs 1/*T* plot, thereby allowing a determination of <*U*>. The linear behavior shows that the electrical conduction process in these hydrated membranes is a thermally activated process. The average depth of the barrier for enabling charge transport through the membrane, i.e., <*U*>, is calculated from the slope of linear fits and their values are shown in the figure for the different hydrated natural fibers. Note all these measurements have been performed on the fibers maintained in their native state. (**b**) A mechanistic representation of thermally activated charge transport. The image shows a charge trapped inside a potential well. The figure shows the tilted potential well when a voltage *V* (or an electric field *E*) is applied, and the charge hops out and drifts preferentially in the direction of the electric field thereby leading to thermally activated charge transport.

Transport in biological membranes also depends on the specific chemical nature of the fibers. A reason we study materials with chemically distinct macromolecules, like, silk cocoon membranes, human hair, corn silk and jute, is to check if the charge transport properties differ with the chemical composition of these materials. We have confirmed in the Extended Data Figs 2 and 3, that the nonlinear, hysteretic nature and the scaling properties of *IV* (similar to Fig. 1) are seen in different hydrated membranes of natural fibers which are chemically distinct as well as in different samples of the same material.

**Figure 3:**
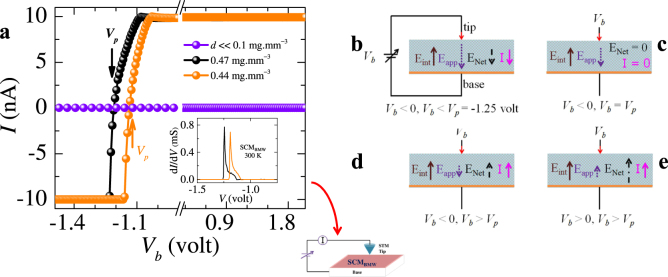
STM study of SCM_BMW_. STM study is done on a piece of hydrated SCM_BMW_ with dimension: 9 mm (length) × 9 mm (width) × 0.5 mm (thickness). (**a**) Here we have chosen the symbol size to be comparable to the size of the error bars. Tunneling current (*I*) between an atomically sharp STM tip and the surface of a hydrated SCM_BMW_ membrane is measured as a function of bias voltage (*V*_*b*_). The figure adjoining (**a**) marked by a curved downward pointing arrow shows the schematic of our STM measurement setup, where the tunneling current *I* is measured between an atomically sharp STM tip and the surface of a hydrated membrane (SCM_BMW_) placed on an electrically conducting base. The bias voltage is applied between the STM tip and the conducting substrate. A tunneling current is established when the Fermi level of the metallic STM tip coincides with available molecular orbital energy levels in the hydrated membrane (see Extended Data Fig. 4). The tunneling current *I* vs *V*_*b*_ is measured for SCM_BMW_ membrane at different water concentration *d* (the *d* values are shown in the plot). All STM measurements were performed at room temperature. Note *I* is zero when *d* is below the critical value (*d*_*c*_ ~0.1 mg.mm^−3^). At different values of *d*, when *V*_*b*_ is changed from −1.25 V to positive values, the tunneling current *I* increases from −10 nA to +10 nA by crossing zero at *V*_*b*  _* = V*_*p*_ = −1.25 V (shown with arrow). The *V*_*p*_ reduces to zero as *d* decreases. The change in the sign of the tunneling current *I* at negative value of *V*_*b*_ we see in (**a**) is unusual. Inset of (**a**) shows d*I*/d*V* vs *V*_*b*_ for different *d*. See Extended Data Fig. 4 for an explanation of the density of states in the medium. (**b–e**) Schematics to understand the behavior of sign switching of the STM tunneling current at negative bias value *V*_*p*_. We consider the membrane develops an internal electric field ${\overrightarrow{E}}_{\mathrm{int}}$ when subjected to a bias voltage. The ${\overrightarrow{E}}_{\mathrm{int}}$ is assumed to be uniform. When *V*_*b*_ *<* *V*_*p*_ and *V*_*b*_ < 0 (see **b**), the net electric field ${\overrightarrow{E}}_{net}={\overrightarrow{E}}_{app}+{\overrightarrow{E}}_{\mathrm{int}}$ is in the direction of ${\overrightarrow{E}}_{app}$ as it is larger than ${\overrightarrow{E}}_{\mathrm{int}}$. Near *V*_*b*_ = *V*_*p*_ = −1.25 V (Fig. **c**), the ${\overrightarrow{E}}_{app}$ becomes equal in magnitude and oppositely directed to ${\overrightarrow{E}}_{\mathrm{int}}$ therefore ${\overrightarrow{E}}_{net}$ → 0 and consequently the tunneling current measured in the STM approaches zero (arrow position in **a**). As *V*_*b*_ > *V*_*p*_ = −1.25 V, ${\overrightarrow{E}}_{net}$ becomes positive and remains positive with further increase in *V*_*b*_ (**d** and **e**) and hence the *I* changes sign from negative to positive. In these schematics, the orange line (base) represents the contact of SCM_BMW_ with the stub of STM sample holder where we are applying *V*_*b*_ and tip represents STM tip (in all Fig. from **b–e**).

### Temperature dependence of bulk electrical conductivity of these natural membranes

We explore below the nature of charge transport mechanism through the temperature dependence of electrical conductivity of different natural fibrous membranes and show the common features of this transport mechanism which is shared across different class of hydrated membranes. The linear behavior in Fig. 2a shows the measured electrical conductivity of different hydrated membranes obeys the Arrhenius like thermally activated behavior of the form, $\sigma \propto \exp (- < U > /{k}_{B}T)$, where <*U*> is mean energy scale and *k*_*B*_ is the Boltzmann constant.

The < *U* > represents the energy barrier which an electron in the membrane must overcome to conduct current across the membrane in the presence of a biasing electric field (see a schematic of the mechanistic view of thermally activated transport shown in Fig. 2b). The slope of the linear fitted line (solid lines) in Fig. 2a gives an estimate of <*U*> ~0.22 eV for both hydrated silk cocoon membrane and human hair and about 0.12 eV for corn silk. Interestingly the jute fiber which is a pure carbohydrate polymer has a low <*U*> value i.e., 0.11 eV and it also has the highest electrical conductivity (*σ*) amongst the natural fibers we have studied. We see that these membranes composed of natural fibers with distinct chemical composition, exhibit similar thermally activated electrical transport properties and similar *IV* characteristics (recall Fig. 1, Extended Data Figs 2 & 3 and Fig. 2). As the order of magnitude of <*U*> is similar across these different membranes, it suggests that the charge transport mechanism is not dependent on the specific chemical nature of the molecular scaffolds present in these hydrated membranes, viz., the scaffolds of Protein − H_2_O polymer (present in silk cocoon and human hair), Carbohydrates − H_2_O polymer (present in jute) and Protein/Carbohydrate hybrid − H_2_O polymer (present in corn silk) complexes. Our results show that presence of water in these molecular scaffolds is necessary for ensuring charge transport in these membranes. Figures 1 and 2 suggest the interesting possibility of controlling the electrical conductivity of bio-inspired native electrically active materials through the choice of natural membrane type, temperature and hydration levels.

### Local electrical characterization of biological membrane (SCM_BMW_) using STM

The bulk electrical transport studies show the presence of potential wells, which trap the charges in the hydrated biological membranes (see Fig. 2a). We now explore the microscopic local electric potentials present in these hydrated fibers which trap these charges. For this purpose, we use the scanning tunneling microscope (STM) to apply a local electric field using a bias voltage (*V*_*b*_) between an STM tip and the surface of the material being studied (hydrated SCM_BMW_ membrane in our case). Information about the local electrical properties of the material is revealed by measuring the electron tunneling current established between the atomically sharp STM tip and the surface of a hydrated silk cocoon membrane (SCM_BMW_) as a function of *V*_*b*_ (see Methods). In recent times, STM based tunneling current *IV* measurements have become popular to probe molecular electronic levels of molecular break junctions^29,31–34^. In our measurements instead of fabricating nanoscale junction between a STM tip and a molecule, we study the tunneling *IV* characteristics of the native hydrated membrane. We would also like to mention that our measurements are unlike earlier STM studies, which have been conducted on de-hydrated and usually denatured molecular junctions^29,33–35^. Figure 3a shows the STM tunneling current increases from negative values ~ −10 nA at a negative voltage bias. At the characteristic applied bias *V*_*p*_ = −1.25 V (see the arrow in Fig. 3a) the tunneling current crosses zero and switches sign to become positive. The value of *V*_*p*_ shifts towards zero with decreasing *d*. The tunneling current in Fig. 3a changes sign over relatively narrower *V*_*b*_ range compared to the broad and gradual change found in studies on molecular junctions^29,33–35^. The typical tunneling current in non-hydrated molecular junctions is few nanoAmps, which is similar to our measurements, at comparable *V*_*b*_. A tunneling current is established between tip and membrane whenever vacant energy levels get aligned with occupied energy levels, present across the tunneling gap (see Extended Data Fig. 4). In STM studies, d*I*/d*V* is a measure of density of states inside the material, which are available for charges to tunnel from an STM tip into the hydrated membrane material or vice versa. The d*I*/d*V* plot in Fig. 3a inset shows a significantly enhanced density of states available near *V*_*p*_, while away from *V*_*p*_ the d*I*/d*V* is uniform and low. Significance of these results is presented in the discussion section.

**Figure 4:**
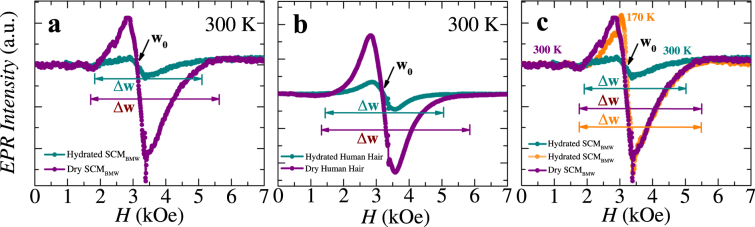
EPR study of different biological membranes. (**a**,**b**) First derivative of EPR absorption spectra is shown as a function of applied magnetic field (*H*) at 300 K for dry (purple) and hydrated (dark cyan) SCM_BMW_ and human hair. (**c**) In this figure we have shown low temperature (170 K) (orange color) EPR absorption spectra for SCM_BMW_ which is similar to the signal of dry SCM_BMW_ (purple color data). The EPR measurement on these membranes clearly show change in the width and intensity of the signal with addition of water. Note that in all figures, we have chosen the symbol size comparable to the size of the error bars.

### Identifying the nature of charge carrier in hydrated biological membranes using EPR

In bulk electrical transport investigations, the conventional Hall measurement is used to determine the nature of the charge carriers present in a material. In Hall measurements, a transverse (Hall) voltage appears across a material in a direction which is perpendicular to the plane of the current and an applied magnetic field. However, in our hydrated natural membranes it is difficult to make a transverse Hall voltage contacts. Furthermore, in these membranes due to the presence of a complex crisscrossing network of fibers, the conducting paths of charges are unconventional and hence a Hall measurement would be difficult to interpret. Therefore, to determine the nature of the charge carriers, instead of using transverse Hall measurements, we use the EPR measurement. Figure 4 shows the first derivative of EPR absorption spectra (see Methods) of SCM_BMW_ and human hair membranes in dry and hydrated state (see Extended Data Fig. 5 for jute fiber). The derivative of the EPR absorption spectra of dry and hydrated samples of SCM_BMW_ and human hair in Fig. 4, a and b show that with hydration the center of the EPR absorption spectrum (vertical arrow, w_0_) does not shift, although there is a significant change in the width of the spectrum (Δw). The hydrated samples of these fibers (SCM_BMW_, hair and jute) show, a narrower spectrum and the EPR intensity also reduces when compared to dry fibers spectrum.

**Figure 5:**
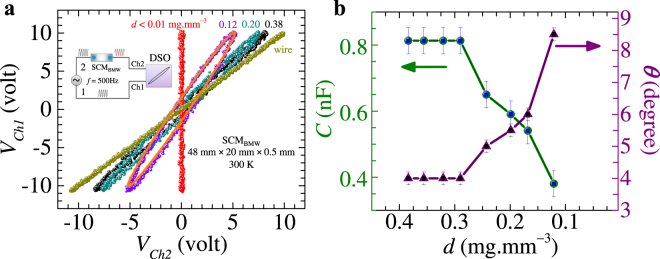
Dielectric property of SCM_BMW_. Measurement is done on a piece of SCM_BMW_ of the following size: 48 mm (length) × 20 mm (width) × 0.5 mm (thickness) using Yokogawa DL 9000 series DSO. Electrical contacts are made at the two ends of the sample (distance between contacts ≈ 46 mm). (**a**) In this figure we have chosen the symbol size comparable to the size of the error bars. Lissajous curves are shown (violet, dark cyan and black color data) for different water concentration *d* (values are shown in the plot). Depending on *d*, the shape of Lissajous curve changes. The inset shows the schematic of the experimental set up. (**b**) Figure shows dependence of phase difference *θ*, (right axis), between the signals from channel 1 and channel 2, on water concentration *d* (bottom axis). The behavior of capacitance, *C* (left axis) versus *d* is also shown. One observes the *C* of the membrane drops as the water content *d* < 0.1 mg.mm^−3^.

Similar line width narrowing with hydration in silk cocoon membranes has been seen in NMR studies (see figure related to NMR data in ref.^1^). The narrowing down of the line width is attributed to the hydration induced enhancement in the mobility of molecular components in the macromolecules of silk fibroin, as deduced from neutron scattering experiments^36^. For both SCM_BMW_ and human hair, w_0_ is located between 3.1 to 3.2 kOe. The location of w_0_ is comparable with earlier EPR data on SCM_BMW_^37^. From w_0_ the calculated gyromagnetic ratio (*g*) is between 2.008 to 2.010 (see Extended Data, Table 1) implying that the charge carriers are electrons and not protons (the absolute value of the *g* for electron is ~2.0023, while for protons it is ~5.586) in all these natural fibers. As the measured gyromagnetic ratio represents an electron like charge rather than a proton, we therefore exclude the possibility that, charge transport in these hydrated membranes is via protonic, Zundell ion ((H_5_O_2_)^+^) complexes formed by reconfiguring and distorting *H*-bonds in H_2_O molecular clusters^8,9,11^. The charge carriers in these systems are fundamentally electronic in nature. In Fig. 4c we show that the EPR spectra of a frozen hydrated SCM_BMW_ measured at 170 K (−103 °C, orange curve). Note that its EPR line width (Δw) at 170 K is identical to that of a dry SCM_BMW_ at room temperature. We will discuss these results in the discussion section.

### Dielectric properties of hydrated SCM_BMW_

Our STM measurements (in Fig. 3) showed the presence of local internal electric field in the hydrated SCM_BMW_, whose value depends upon the water concentration within the membrane. The presence of an internal electric field suggests dielectric property of the hydrated silk cocoon membrane. To investigate the dielectric properties of the hydrated SCM_BMW_ membranes, we measure the phase difference between two sinusoidal electrical signals, *V*(*t*) = *10sin*(*ωt*) (volt), with $\omega =2\pi (500\,{\rm{Hz}})$ sent down to two shielded BNC cables (labeled 1 and 2 in the schematic of Fig. 5a). The signals from cables 1 and 2 are measured on the channels, Ch1 and Ch2 of a digital storage oscilloscope (DSO), respectively with a hydrated SCM_BMW_ membrane placed in the path of cable 2 (see the inset of Fig. 5a). Figure 5a shows the Lissajous curves obtained by plotting ${V}_{Ch2}(t)$(Voltage from cable 2) versus ${V}_{Ch1}(t)$ (Voltage from cable 1, without SCM_BMW_ in its path). Note that, when the cable 2 is electrically continuous (i.e., cable 2 is directly connected to the DSO without the SCM_BMW_ placed in the path of cable 2) then *V*_*Ch1*_(*t*) = *V*_*Ch2*_(*t*) = *10sin*(*ωt*) (volt), and we observe a straight line with a 45° slope (dark yellow data, labeled as wire) in Fig. 5a. When the SCM_BMW_ membrane is completely dry, viz., *d* < 0.01 mg.mm^−3^, as ${V}_{Ch2}(t)$ ~0, therefore we obtain the vertical (red) line in Fig. 5a. At intermediate levels of water content (*d*) in the SCM_BMW_ we obtain elliptical shaped Lissajous curves, as the membrane introduces a phase difference (*θ*, a fitting parameter) between the electrical paths through cables 1 and 2. These Lissajous curves are fitted using ${V}_{Ch1}(t)={a}_{1}sin(\omega t)$, where ${a}_{1}=10$ V and ${V}_{Ch2}(t)={a}_{2}sin(\omega t+\theta )$ where ${a}_{2}$ varies from 8.5 V to 5.5 V as *d* of the hydrated SCM_BMW_ changes from 0.38 to 0.12 mg.mm^−3^ (see orange fitted curve through violet data points in Fig. 5a). The *θ* value determined from this fitting, is plotted as a function of *d* in Fig. 5b (see right axis).

For the hydrated SCM_BMW_ membrane we model the behavior of the phase difference *θ* w.r.t. *ω* as an electrical circuit with bulk equivalent resistance (*R*) *-* capacitance (*C*), where the phase difference at *ω* is $\theta ={\tan }^{-1}(\frac{1}{\omega RC})$. Using this formula and the *R*(*d*) behavior from Fig. 1a, the *θ*(*d*) plot of Fig. 5b is converted into *C*(*d*) behavior (see left axis in Fig. 5b). The capacitance of the hydrated SCM_BMW_ is found to remain constant at about 0.8 nF for a water content *d* > 0.3 mg.mm^−3^. As the membrane dries, we see that *C* drops rapidly down to 0.4 nF as *d* → 0.1 mg.mm^−3^. To determine the *C* independently, we construct a parallel plate capacitor by placing a piece of hydrated SCM_BMW_ (*d* ~0.2 mg.mm^−3^) between the two Cu plates of size 1 cm × 1 cm (see the actual picture of the device in Extended Data Fig. 6). By using the Lissajous curve method described above, we determine a capacitance of hydrated SCM_BMW_ ~3 × 10^−10^ F (which is consistent with the values in Fig. 5b). Using the expression for a parallel plate capacitor, $C=\frac{{\varepsilon }_{r}{\varepsilon }_{0}A}{l}$ where, ${\varepsilon }_{r}$ is the dielectric constant, *ε*_*0*_ is the vacuum permittivity, *A* (1 cm × 1 cm) is the area and *l* (0.5 mm) is the thickness of the SCM_BMW_, we estimate a high ${\varepsilon }_{r}$~ 170 for the hydrated SCM_BMW_. The effective dielectric constant, ${\varepsilon }_{r}$ of the hydrated SCM_BMW_ is almost double than that of free water which is about 80.8. The high ${\varepsilon }_{r}$ suggests a high dielectric susceptibility, $\chi ={\varepsilon }_{r}-1$ ~169, implies that the SCM_BMW_ membrane with hydration, gets significantly polarized, electrically. This internal polarization of the membrane is responsible for the local electric fields measured in STM.

**Figure 6:**
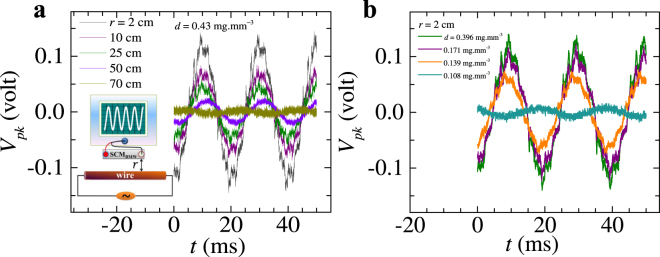
Electric field signal pick up by hydrated SCM_BMW_. For this pick up measurement we have taken a sample of following size: 48 mm (length) × 20 mm (width) × 0.5 mm (thickness). For measurement we have used Yokogawa DL 9000 series DSO. Electrical contacts are made at the two ends of the sample (distance between contacts ≈ 46 mm). (**a**) Figure shows the 50 Hz AC signal picked up by hydrated SCM_BMW_ kept at different distances. Inset shows the schematic of experimental set-up. (**b**) Figure shows the dependence of water concentration on the sensing capability of hydrated SCM_BMW_. Here we see that when *d* < 0.1 mg.mm^−3^ SCM_BMW_ is not able to pick up the AC signal kept at distance of 2 cm from the wire. Note that in both the figures, the typical level of fluctuation in the data is of the order of 2 mV.

### Electric field sensing properties of SCM_BMW_

As seen above, due to the relatively high dielectric constant of the SCM_BMW_ it is likely to make these membranes sensitive to electric fields present in the environment around the membrane. We record the voltage generated across a SCM_BMW_ which is placed at different distances (*r*) from the surface of a shielded copper wire carrying an AC current 0.5 (r.m.s) at 50 Hz. We observe in Fig. 6a that in this non-contact configuration, the hydrated silk cocoon membrane shows a time varying voltage signal pick up (*V*_*pk*_) across the voltage contacts on the membrane, whenever an AC current flows through the wire (kept at a distance *r*). The *V*_*pk*_(*t*) signal has a frequency of 50 Hz and the pickup signal strength decreases as the distance *r* between the hydrated silk cocoon membrane and the wire increases. We find that up to a distance of 50 cm from the wire surface, the hydrated SCM_BMW_ is capable of picking the electric field modulations present in the environment due to an AC current carrying wire. Note that, we ensured that during these measurements no other current carrying wire was present in a radius of 1 meter around the hydrated membrane (see Supplementary Material Video 1).

In Fig. 6b, we show that SCM_BMW_ sensing property is dependent on water concentration. We keep the SCM_BMW_ at fixed distance from an AC current (50 Hz) carrying wire. We see that the pickup signal strength decreases with *d* (all the while *r* is fixed). At low water concentration of *d* < 0.1 mg.mm^−3^, the membrane is not sensitive to pick up the 50 Hz signal. In a separate measurement (see Extended Data Fig. 7) we find that beyond ~1 kHz, the voltage picked up by the silk cocoon membrane drops.

The property of the hydrated silk cocoon membranes to pick up low frequency electric field modulations from the environment makes it a lucrative option to explore if it is sensitive to bio-rhythmic signals when placed in contact with a human skin.

**Figure 7:**
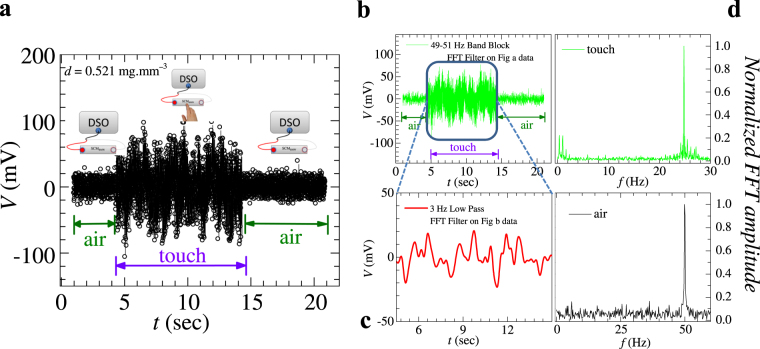
Performance of SCM_BMW_ as sensor. For this pick up measurement we have taken a sample of following size: 48 mm (length) × 20 mm (width) × 0.5 mm (thickness). Electrical contacts are made at the two ends of the sample (distance between contacts ≈ 46 mm). For measurement we have used Yokogawa DL 9000 series DSO. (**a**) At a given water concentration (0.521 mg.mm^−3^), signal picked up by the hydrated SCM_BMW_ membrane is shown. The voltage time series *V*(*t*) signal measured across hydrated SCM_BMW_ membrane can be divided into two regions. The portion of *V*(*t*) marked as ‘air’ is when no human skin is in contact with the hydrated SCM_BMW_ (see left and right side schematics in **a**). Here the *V*(*t*) signal contains the time varying electrical signals picked up from the ambient environment. The middle portion of *V*(*t*) signal marked as ‘touch’ is when the hydrated silk cocoon membrane is placed in contact with human skin (see middle schematic in **a**). (**b**) The *V*(*t*) in **a** is replotted here after applying a 49–51 Hz band block Fast Fourier Transform (FFT) filter (in order to remove signals coming from the nearby electrical wires carrying electrical currents at 50 Hz). (**c**) A 3 Hz low pass FFT filter is applied on Fig. **b** data (only on the squared region) to extract only the low frequency modulations present in *V*(*t*) in that region of *V*(*t*) when the membrane is in contact with human skin. Here we observe low frequency signal below 3 Hz. (**d**) FFT analysis is done on the signal present inside the squared region (viz., when the membrane is in contact with human) of (**b**) (FFT) spectra of only that portion of *V*(*t*) marked touch is shown in (**d**). The FFT shows two peaks in the spectrum, one which is below 3 Hz and other is between 22–28 Hz. This low frequency bunch (<3 Hz) is also present in (**c**). Y-axis has been normalized with the peak value of the FFT amplitude. (**e**) This figure shows FFT signal when hydrated SCM_BMW_ is in air (shown in **a**). Here we see that only 50 Hz frequency signal is present in FFT spectrum which is picked up by nearby current carrying cable. Y-axis has been normalized with the peak value of the FFT amplitude. In **a** and **b**, the typical level of fluctuation in the data is of the order of 5 mV.

We keep a hydrated SCM_BMW_ membrane (*d* = 0.52 mg.mm^−3^) in direct contact with a live human skin and the voltage time series (*V*(*t*)) developed across the two ends of the membrane are recorded on a digital storage oscilloscope (DSO) (see schematic in Fig. 7a). Figure 7a shows how the *V*(*t*) gets modified when the hydrated SCM_BMW_ comes in contact with electric potentials present on the human skin (see *V*(*t*) portion in Fig. 7a marked ‘touch’). By taking a Fast Fourier Transform (FFT) of the portion of the *V*(*t*) in Fig. 7a labeled air, we observe that the spectrum shows a distinct peak only around 50 Hz in Fig. 7e, which is the signal of line frequency picked up from the environment (also see Extended Data Fig. 8). The green curve in Fig. 7b is obtained by applying a 49 to 51 Hz band block filter on *V*(*t*) of Fig. 7a to filter out the line frequency and the red curve in Fig. 7c is obtained by further applying a 3 Hz low pass filter on the filtered green curve (filtering is done only on the portion of the signal present inside the square region in Fig. 7b). Figure 7d is the FFT of the *V*(*t*) spectrum in Fig. 7b, and it shows peaks bunched near 22 to 28 Hz and peaks below 3 Hz (Supplementary Material Video 2). We were especially interested in the structure present in the FFT spectrum below 3 Hz, as such low frequency fluctuations are not produced by conventional electronic source (note the absence of 3 Hz and 22 to 28 Hz peak in the FFT spectrum in Fig. 7e when the hydrated SCM_BMW_ membrane is placed only in ambient air and not touching any human skin). A possible source of these low frequency signals could be rhythmic processes in the human body like heart beats which are in frequency range of 1 to 2 Hz.

**Figure 8:**
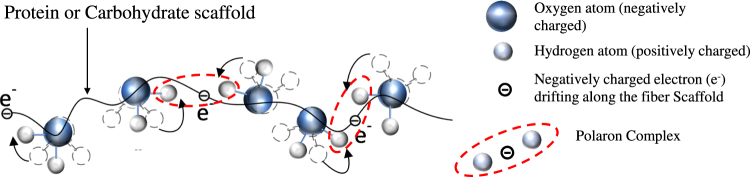
Water mediated hopping of ‘Polaron-like complexes’ on the glassy landscape of fibers. The black wavy line represents the Protein or Carbohydrate molecular scaffold on which water molecules are arranged. The shaded blue color circle represents oxygen (negatively charged) and grey color circle represents hydrogen (positively charged) ends of the water molecule. When an electron is in the vicinity of the water molecule on the molecular scaffold, then the positively charged ends of the water molecule get polarized in the electric field of the electron and it distort slightly towards the electron. The slight shift between the colored schematic of the water molecule and the dashed outline of the molecule has been intentionally done, in order to represent a rotation or distortion of the molecule when an electron (black circle) approaches it. The process of reorientation of the water molecule is shown by curved black arrows. In this process an intermediate Polaron complex gets created (see red dashed elliptical region), where the electron is surrounded by a cloud of positive charge fluctuations created by the positively charged ends of the water molecule distorting towards the electron. The positive cloud attempts to shield the charge of the electron by enveloping the electron within the positive charge. This leads to a reduction in the effective charge of the Polaron. Due to its lower effective charge, the Polaron gets effectively decoupled from the charged local chemical environment on the molecular scaffold leading to a narrowing down of the EPR line width. The above process can only occur in the presence of water.

## Discussion

Our bulk transport measurements have shown that the electrical conductivity of some natural membranes, is poor in their dry state, and it improves with hydration. Local STM measurements show, the tunneling current switches sign (see Fig. 3) at a negative bias voltage *V*_*p*_ instead of zero, signifying the presence of an internal electric field. We estimate the magnitude of this internal electric field to be ${E}_{\mathrm{int}}=\frac{{V}_{p}}{s}$ ~2.5 × 10^3^ V/m, where *s* ~0.5 mm is the membrane thickness (see schematics Fig. 3b–e). The *E*_*int*_ represents the hydrated membrane is significantly electrically polarized. This feature is consistent with our measurement of high dielectric susceptibility ~169 associated with these hydrated membranes. At *V*_*b*_ = *V*_*p*_ = *E*_*int*_.*s* the internal electric field inside the hydrated membrane gets compensated by the applied field (Fig. 3c) and thus the tunneling *I* → 0 at *V*_*b*_ = *V*_*p*_. This STM measurement shows presence of enhanced density of states (larger d*I*/d*V*) around *V*_*p*_. We observe *V*_*p*_ decreases with *d* (Fig. 3a) which suggests the internal electric field (*E*_*int*_) in the membrane decreases with water content. Also note that with shifting *V*_*p*_ the peak density of states also shifts with *d* (Fig. 3a inset). When the water content of the SCM_BMW_ falls well below 0.1 mg.mm^−3^ then we do not find any evidence of *V*_*p*_ and d*I*/d*V*→ 0 (i.e., there are no available electronic states for electrons to tunnel into). These results suggest that water controls the generation of local electric fields through polarization produced inside the membrane. This electric field is important for charge conduction in these membranes as we see there are available density of states at *V*_*p*_, which changes with the water content in the membrane.

Through EPR measurements, we identify electrons as the basic charge carrying entity in these natural membranes. These studies also showed an unusual variation in the EPR line width with hydration and temperature. To investigate this issue further, we recall the behavior of the EPR line width (Fig. 4). We found that the EPR line width narrows down with increasing water concentration in the membrane. Also with lowering of temperature as the water in the membrane freezes, the EPR line width broadens and becomes equal to that of the dry membrane at room temperature. The EPR line width changes with the coupling strength of the electron with its local environment. The coupling of the electron to its environment is via spin - spin interaction or via Coulombic interactions. If electric charges in the hydrated membrane were completely free electrons, then one would not have observed changes in the EPR line width. A broadening of the EPR line width when the fibers are dry (*d* < 0.1 mg.mm^−3^), suggests that electrons are strongly coupled to the complex chemical environment in these natural fibers. The narrowing down of the EPR line width with hydration suggests the electron begins to decouple from its complex charged local chemical environment in the presence of water.

From these EPR results it appears that free electron transport is absent in these hydrated, dielectric membrane of the natural fibers. We argue for an alternative mechanism of electron transport in these hydrated fibers: From Fig. 1a recall the sharp increase in resistance below a critical water concentration *d*_c_ = 0.1 mg.mm^−3^. A *d*_c_ = 0.1 mg.mm^−3^ corresponds to a density (*n*_*c*_) of 3.35 × 10^21^ water molecules.cm^−3^. Using *n*_*c*_, we make a crude estimate that at *d*_*c*_ the average spacing between water molecules on the molecular scaffolds in the membrane is *ξ*_*c*_ ~*n*_c_^−1/3^ ~6.68 Å. It turns out that this estimate of *ξ*_*c*_ is similar to the repeat distance of 7 Å for the R-group in the beta-keratin protein chain of silk cocoon^25^. Based on the correlation that resistance diverges for, *d* < *d*_*c*_, and *d*_*c*_ is associated with the average water molecule separation becoming of the order of a characteristic length scale on the molecular scaffold of the silk cocoon, suggests that water molecules decorate the molecular scaffold of a natural fiber and they affect the electrical conductivity of the fibers. This presence of water molecules decorating the Protein scaffold makes the water-Protein scaffold complex highly susceptible to electric polarization, as the polarizable water molecules can reorient and distort in an electric field. In fact earlier studies on enhancement in dielectric constant of hydrated Proteins have been attributed to the formation of Protein-water complex^10^. On the molecular scaffold decorated by water molecules, an electron traversing along the scaffold, electrically polarizes the hydrated membrane by deforming the water molecules in its local environment (see schematic in Fig. 8). The above feature of the hydrated natural fibers developing additional degree of freedom due to the ability of water molecules to distort, is consistent with observations of increased molecular mobility seen in Neutron scattering^36^ experiments, which are considered to narrow down NMR line widths^1^ in hydrated silk fiber.

The electric field of an approaching electron polarizes (see Fig. 8) the water molecules momentarily distorting the positively charged hydrogen (shaded grey) end distorting towards the electron. This leads to the formation of an intermediate composite state, viz., a Polaron complex (see schematic in Fig. 8). The Polaron^38^ complex is an electron surrounded by cloud of positive charge fluctuations generated by the distorting hydrogen ends of water molecules. The creation of the positive charge cloud in turn attracts the electrons in its vicinity and in turn facilitates charge conduction along the molecular scaffold. Note that Polarons mediated charge transport mechanism is a well-known mechanism in some condensed matter systems, like, the high resistance dielectric organic polymers^39,40^. A Polaron requires distortion of the water molecules, therefore the characteristic energy of the Polaron can be considered to be ~$\hslash \omega$, where *ω* is a characteristic vibration frequency in this system. The energy of 0.2 eV (see typical <*U*> values in Fig. 2a) we propose is associated with the energy of a Polaron. This energy scale of 0.2 eV is in the same range of as the intramolecular vibrational transitions in the mid IR regime of water molecule. This observation supports our view that charge transport along the molecular scaffold occurs by exciting vibrational modes (distorting water molecules) in the water molecules, whose energies are in the range of 0.2 eV. It may also be mentioned in passing that 0.2 eV Polarons have been reported in other non - biological systems^41^.

We now show that the reduced effective charge on the Polaron can also be estimated from our experimental results in the following way: Recall our STM measurements showed the presence of a local internal field inside the hydrated membrane which corresponds to a characteristic internal potential difference of magnitude, |*V*_*p*_| ~ 1.25 V (see Fig. 3). Inside the hydrated membrane, trapping of charges by the local internal electric field is related to the activation barrier <*U*> ~0.2 *e*V, determined from the temperature-dependent bulk conductivity measurements of Fig. 2. For a Polaron with net effective charge *q*, in order for it to hop it does work against the internal electric field, i.e., it does a work *q*|*V*_*p*_|, with < *U* > ~ *q*|*V*_*p*_|. Using the values of < *U* > and |*V*_*p*_|, we estimate the Polaron has a net effective charge *q ~*0.16*e*, where *e* is the charge of a free electron. Thus shielding of the free electrons charge by the positive charge cloud in the Polaronic state, significantly reduces its net charge compared to that of a free electron. An indirect evidence of reduction of the effective electrical charge on the charge carrying entity in a hydrated membrane is hydration induced narrowing down of the EPR line width. A Polaron with lower effective charge is electrostatically more weakly coupled to the local charged environment on the molecular scaffold. Hence a narrower EPR line width results (Fig. 4a–c) when the membrane is hydrated. Recall in Fig. 4c we had shown that the EPR spectra of a frozen hydrated SCM_BMW_ measured at 170 K (−103 °C, orange curve). Note that its EPR line width (Δw) at 170 K is identical to that of a dry SCM_BMW_ at room temperature. At low temperature the water molecules freeze thereby reducing their degree of freedom on the molecular scaffold and hence, they cannot distort easily to create a Polaron when an electron approaches. Therefore, as a Polaron cannot form, hence ineffective shielding of the electron’s charge at low temperature enhances the coupling of the electron to its local charged chemical environment which leads to a broadening of the EPR spectra in the frozen state back to a level which is comparable to that in the dry state of the membrane (Fig. 4c). Apart from temperature, enhanced electrostatic repulsion between water molecules at higher water molecule density also hampers their ability to distort or reorient water molecules. Hence, high concentration of water molecules in the membrane would also inhibit formation of a Polaron (as distortions of water molecules are not favored) and hence *R* increases at high water concentrations (*d*) (see Fig. 1c). The formation of Polaron leads to enhanced density of states found in Fig. 3a inset for the hydrated natural fiber (also see schematic in Extended Data Fig. 4). Due to the electrically polarizable nature of the hydrated medium of the natural fiber, application of *V* affects the dielectric medium and also its conductivity. Therefore, it is not surprising to observe the nonlinear nature of *IV* as shown in Fig. 1b.

Furthermore, an outcome of our study is that these hydrated electrically polarizable membranes are potentially useful as sensors to detect electrical fields in the environment or in live human tissues (see Figs 6 and 7). From our measurements we know the typical membrane resistance is ~10 MΩ with a capacitance ~0.1 nF. This gives a typical *RC* time constant ~1 msec (or (RC)^−1^ ~1 kHz) for these membranes. Hence the typical band-width of these bio-membrane sensors is from low frequencies upto 1 kHz which is consistent with our measured cut off frequency (see Extended Data Fig. 7). This gives a unique advantage to these sensors, as they detect electric field modulations in the low frequency regime, whereas other conventional sensors in this regime have low sensitivity due to *1/f* noise considerations.

## Conclusions

In conclusion, we have uncovered the important effect that water plays in not only producing dielectric polarizability in natural fibers but also helps in mediating electron transport across the fibers. Due to the enhancement in their dielectric polarizability, the hydrated natural fibers develop ability to sense electric fields. This property makes these hydrated natural membranes potentially useful for application as sensors for bio-rhythmic processes which are associated with low frequency electric field modulation. Through our present study performed on at least ten samples of silk cocoon membranes, human hair, jute and corn-silk fibers, we have uncovered features of electron transport in these hydrated bio-polymers. However, detailed future studies are needed in this direction on a wider class of bio-membranes, to establish the general features of electrical conductivity across a wide variety of hydrated bio-polymers. While our STM measurements with a sensitivity to detect electric fields of about 0.5 V/m, have revealed the presence of local electric fields (~10^3^ V/m) inside the hydrated silk cocoon membrane, its spatial distribution and fluctuation in time are still undetermined. These properties need to be mapped in detail on the hydrated silk protein scaffold in its native state in order to establish the details of the Polaronic mechanism. We also need to have high resolution imaging of the molecular fabric of the hydrated scaffold at different temperatures. Our method to measure the dielectric property of these membranes, is viable for measurement of dielectric properties as a function of water content and varying temperature. However, the systems measurement sensitivity is limited to about 0.1 nF capacitance, and the technique cannot determine changes in the dielectric properties of the material at a local level or if the quantity of the material being probed is very small. In the future we believe a better understanding of the charge transport mechanism in natural fibers will have potential ramifications for understanding the behavior of electrically active biological tissue.

## Methods

### Electrical Transport Measurement

Electrical measurements are performed at room temperature and pressure using Keithley 2400 source meter. The membrane of *Bombyx mori* silk cocoon (SCM_BMW_) is first cut into small rectangular pieces (size of the membrane is mentioned in the figure captions in main manuscript) and then dipped in deionized water to hydrate the membrane (the purity of distilled water is 2 ppm i.e., there are 2 mg of dissolved solids in 1 kg of water). Electrical contacts are made at the two ends of the membrane. Contacts are made with aluminum (Al) and copper (Cu) wire at two ends (we have also tried with Cu-Cu contacts and electrical characteristics were same in both cases). See section on Image of samples with electrical contacts in Supplementary Information.

For making hair, jute and corn-silk membranes we have used a collection of hair, jute and corn-silk fibers and electrical contacts are made at the two ends of these membranes. Contacts are made with aluminum (Al) and copper (Cu) wire at two ends. Measurements have been done with four probe and two probe. No difference was found between the four probe and two probe measurements as the resistance of the medium is very high. All transport measurements reported in the paper are two probe.

We measure resistance (*R = V/I*) of these membranes as a function of its water content (*d*) by initially hydrating the samples and then allowing them to dry at 300 K. To get the information about water content in the membrane, first, we measure the weight of dry sample. After hydrating the membrane with water we put it on a weighing machine and noted the weight after a fixed interval of time. After subtracting these values from the dry weight we get the water concentration (*d*) in membrane by determining the weight to the volume ratio.

### Scaling of *IV* plot

To check if the nature of the hysteresis in the *IV* curve remained unchanged with varying water content, we scaled them in the following way. Each forward branch of *IV* (viz., we have measured *I* as *V* is swept from a −ve to a +ve value and back to –ve value) in Fig. 1b is fitted to a linear form, *I* = *<R*>^−1^*V*, where <*R*>^−1^ is the mean slope of the *IV* curve (note the analysis is independent of the use of the forward or reverse *IV* branch). Then each *IV* curve is divided by the mean value of the slope.

### STM (Scanning Tunneling Measurement)

The point *IV* measurements of the hydrated silk cocoon is carried out in a Quazar Technologies made room temperature scanning tunneling microscopy system (STM) (NanoRev. 4.0). In our measurement we use an atomically sharp tip fabricated from a Pt-Ir alloy (using electrochemical etching process). The hydrated membrane SCM_BMW_ is mounted in the STM on a gold coated metallic stub (sample holder) and the bias voltage, *V*_*b*_, is applied between the metallic stub and the Pt-Ir tip which is kept at ground potential. When the tip is not touching the membrane and there is a gap of few nm between the STM tip and the surface of the SCM_BMW_ membrane, we observe no appreciable tunnel current. For measuring any appreciable tunneling current we do the following: while maintaining a finite bias voltage *V*_*b*_ between the STM tip and the metallic stub on which the SCM_BMW_ membrane is placed, the STM tip is first lowered towards the surface of the membrane until the tip touches the membrane. At this point a large current is observed. We then begin retracting the tip so that the tip begins leaving the contacted region in the membrane in steps of size 2 Å. During this process at every retracted position, the tip is held stationary and in this position the tunneling current *I* versus bias voltage *V*_*b*_ curve is measured. The process of measuring *I* versus *V*_*b*_ curve is continued at every retracted step of the STM tip, until the tunnel current disappears. We find that the STM tip needs to be retracted by about 20 to 30 Å to reach the zero tunneling current state. *I* versus *V*_*b*_ curves shown in Fig. 3a have been obtained just before the tunneling current disappears.

### EPR (Electron Paramagnetic Resonance Measurement)

The unpaired electron of a material and the electronic structure was studied by electron paramagnetic resonance (EPR). Electron has a spin magnetic moment $\overrightarrow{S}=1/2$, with magnetic component ${m}_{s}=\pm 1/2$. When a paramagnetic material with unpaired electrons is placed in an external magnetic field B_0_, due to the Zeeman effect degenerate energy levels of the unpaired electrons are split in two energy level with energy difference $\Delta E={g}_{e}{\mu }_{B}{B}_{0}$, *g*_*e*_ is the Lande g factor and ${\mu }_{B}$ is the Bohr magneton. If we apply an electromagnetic (EM) signal in the presence of a magnetic field, at the resonance condition an absorption peak is observed. This is the central idea of EPR spectroscopy. If *ν* is the applied frequency of the EM signal, then at resonance $h\upsilon ={g}_{e}{\mu }_{B}{B}_{0}.$

EPR measurement is done using “Bruker EMX EPR Spectrometer”. EPR measurement is carried out at a constant frequency of 9.864 GHz and by varying the magnetic field. Sample is placed in the quartz tube. At first magnetic field is varied over the full range from 0 to 8000 Oe. The precise measurement is done close to the peak region in the absorption spectra. To eliminate any geometric effects affecting the measurements, we have made symmetric bundle of the sample. At first we take the measurement of the sample with the quartz tube. Data is plotted after background (quartz) subtraction.

### Online Content

Additional Extended Data display items and Source Data, are available in the online version of the paper; references unique to these sections appear only in the online paper.

## Electronic supplementary material

Supplementary information

Supplementary Video 1

Supplementary Video 2
