# Supplementary material for: Water mediated dielectric polarizability and electron charge transport properties of high resistance natural fibers
Source: Sci Rep. 2018 Feb 9;8:2726. doi: 10.1038/s41598-018-20313-4 (PMC5807519; doi:10.1038/s41598-018-20313-4)
Supplement: Supplementary file 1 — Supplementary information [file 41598_2018_20313_MOESM1_ESM.pdf]

Water mediated dielectric polarizability and electron charge transport properties of high resistance natural fibers

Ankit Kumar[1], Amit Jash[1], Amarish Dubey[2], Alok Bajpai[3*], Deepu Philip[2,4], Kalpana Bhargava[5], Sushil K Singh[5], Mainak Das[2,6+], S. S. Banerjee[1§]

[1]Department of Physics, Indian Institute of Technology, Kanpur 208016, Uttar Pradesh India

[2]Design Program, Indian Institute of Technology, Kanpur 208016, Uttar Pradesh, India

[3]Psychiatrist, Medical Centre, Indian Institute of Technology Kanpur, Kanpur 208016, Uttar Pradesh, India

[4]Industrial and Management Engineering, Indian Institute of Technology Kanpur, Kanpur 208016, Uttar Pradesh, India

[5]DRDO, Timarpur, Delhi 110054, India

[6]Biological Sciences & Bioengineering, Indian Institute of Technology Kanpur, Kanpur 208016, Uttar Pradesh, India

*email: alokbajp@gmail.com

+email: mainakd@iitk.ac.in

§e-mail: satyajit@iitk.ac.in

Extended Data Table 1 | Summary of Electron paramagnetic resonance (EPR) experiment for hydrated and dry SCM_{BMW} and human hair. Here *B* is applied magnetic field in gauss, *d* is water concentration and *g* is gyromagnetic ratio.

	SCM_{BMW} Dry ($d < 0.01$ $mg.mm^{-3}$)	SCM_{BMW} Wet ($d \sim 0.6$ $mg.mm^{-3}$)	Human Hair Dry ($d < 0.01$ $mg.mm^{-3}$)	Human Hair Wet ($d \sim 0.6$ $mg.mm^{-3}$)
B (Gauss)	3510	3516	3512	3514
g	2.010	2.007	2.009	2.008

Observation of asymptotic tail in the water concentration as a function of time

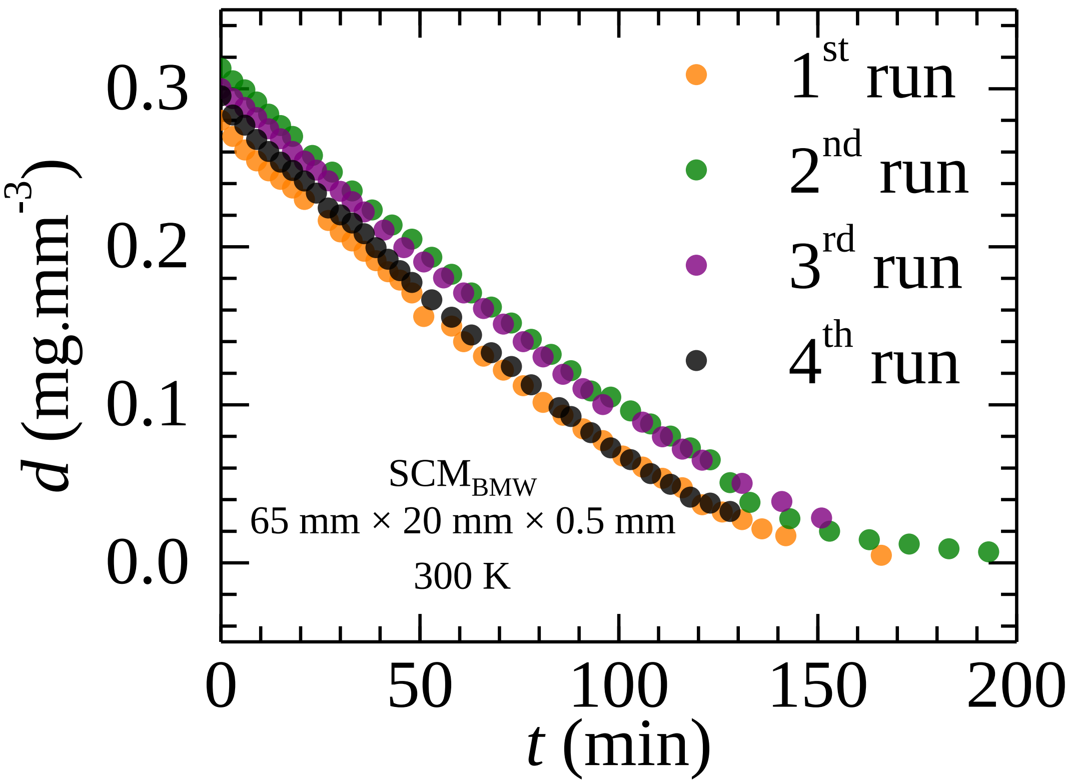

Extended Data Figure 1 | Drying of water from hydrated SCM_{BMW}. Figure shows that drying of water is a linear process only up to a certain time (< 110-120 min). For small *d* values which are less than ~ 0.05 $mg.mm^{-3}$ the $d(t)$ asymptotically approaches zero non-linearly. All our measurement have been performed in a water concentration range in the membrane where $d(t)$ is linear.

Observation of non-linear *IV* and hysteresis in different samples of SCM_{BMW}

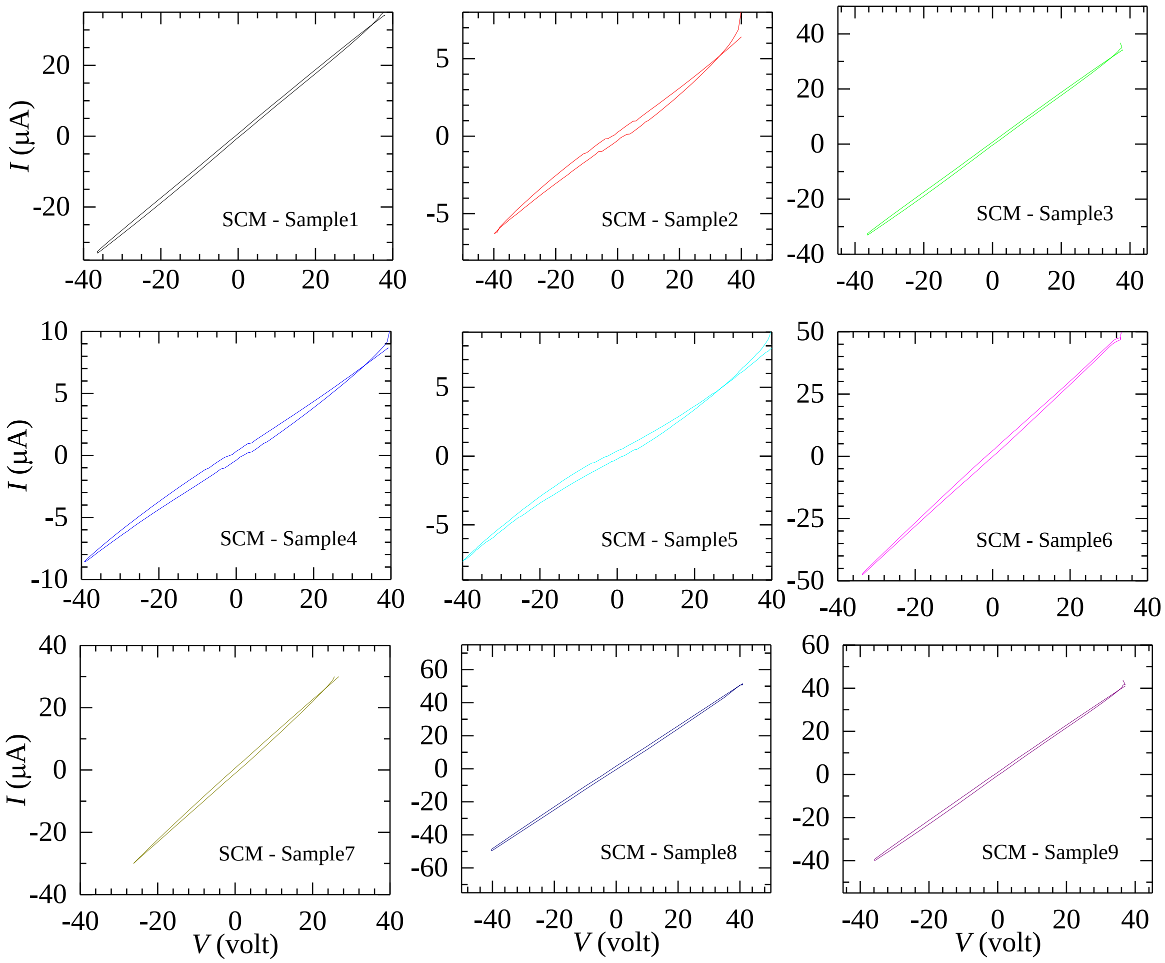

Extended Data Figure 2 | *IV* characteristic of different samples of SCM_{BMW}. We have done *IV* characterization on different SCM samples of same class (Bombyx mori) to check whether this hysteresis in *IV* is sample dependent or not. The figure shows hysteretic *IV* characteristics of samples taken from different silk cocoon membranes where all of them have the same dimensions: 48 mm (length) × 16 mm (width) × 0.5 mm (thickness). The panels above are *IV* measurements performed with different water concentrations in the membranes. Depending on the water concentration we see that the current values are different. The absolute values of the currents can vary from samples to samples however the non-linear *IV* feature and the hysteresis are similar to those shown in Fig. 1 of the main manuscript. From this we infer that the features in the electrical transport we report in Fig. 1 of the main manuscript are not specific to one particular sample of the hydrated silk cocoon membrane.

Scaled *IV* and long time series measurement in different samples of SCM_{AM} and human hair samples

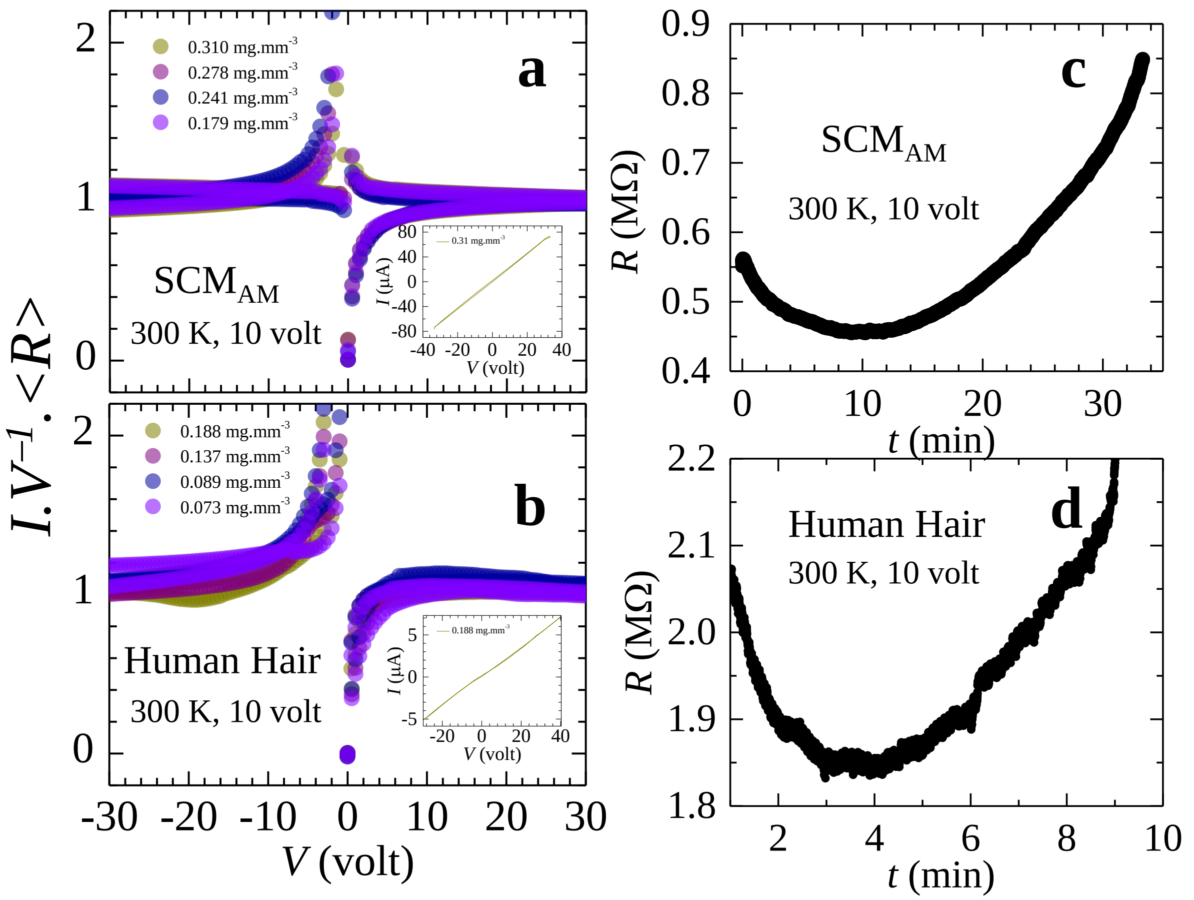

Extended Data Figure 3 | Electrical transport study on different class of SCM and human hair. Insets of Fig **a** and **b** show the non-linear nature of *IV* in hydrated silk cocoon membrane of Antheraea mylitta (SCM_{AM}) and in human hair. The nature of the *IV*'s in SCM_{AM} and human hair are similar as shown in Fig. 1 of the main manuscript, which are results reported on hydrated Bombyx Mori silk cocoon membrane. The main panels of Fig **a** and **b** show the scaled plot (the details of the scaling are described in the Methods section) of $I.V^{-1}.\langle R\rangle$ versus V of hydrated SCM_{AM} (another class of SCM, *Antheraea mylitta*) and human hair (HH). In both cases, we found that all the hysteretic *IV* curves in **a** and **b** at different water concentrations (*d*) get scaled onto one single master curve. This behavior is identical to the *IV* scaling in Fig. 1 of the main manuscript for SCM_{BMW}. This observation implies that the transport properties we have measured are not specific to the chemical nature of these fibers. Fig. **c** and **d** show the behavior of resistance (*R*) with time (which is proportional to *d*) in SCM_{AM} and HH respectively. For this first, we hydrated the samples and then applied 10 volt across it. The *R* vs *t* responses shown here are also similar to that shown for SCM_{BMW} (see the main manuscript Fig. 1c).

Explanation of density of states in STM measurement

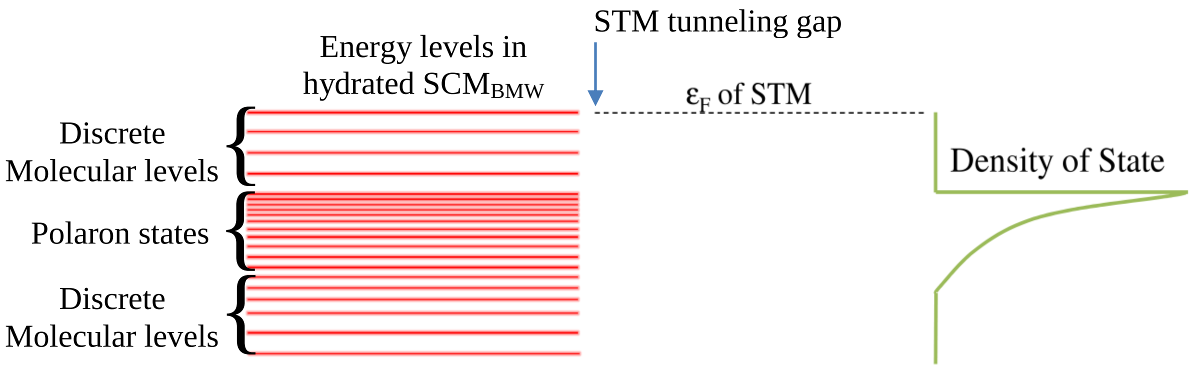

Extended Data Figure 4 | Density of states in SCM_{BMW}. Left most figure above shows our proposed electronic energy level diagram in the hydrated natural fiber membranes. The middle dashed line in the figure represents the Fermi energy level (ε_F) in the STM tip. Tunneling current I is established at different bias voltages V_b when the Fermi energy level in the tip align with different energy levels in the hydrated natural fiber membrane. The relative aligning of different energy levels on either side of the tunneling gap depends on the V_b. The right most curve represents the measured density of energy levels ($\propto$ dI/dV). Here we observe that energy levels are closely spaced close to the middle of energy level diagram. The almost uniformly spaced less dense levels near the top and bottom of the energy diagram are molecular levels in the silk cocoon membrane. The region of high density of energy levels near the middle of the diagram, we propose, are the levels created by the Polaron formation created by local distortions induced during Polaron formation and associated local electric field of the Polaron. The strength of the local electric field associated with the Polaron governs the location of these high density states in the energy level diagram. Hence the location of these high density energy levels changes with moisture concentration (*d*) in the membrane (recall Fig. 3a inset shows the center of the density states shifts with *d*). The energy level shown on the left corresponds to the density of states on the right hand side of the figure which is consistent with the DOS measured in our STM measurements in Fig. 3a inset.

EPR measurement on Jute sample

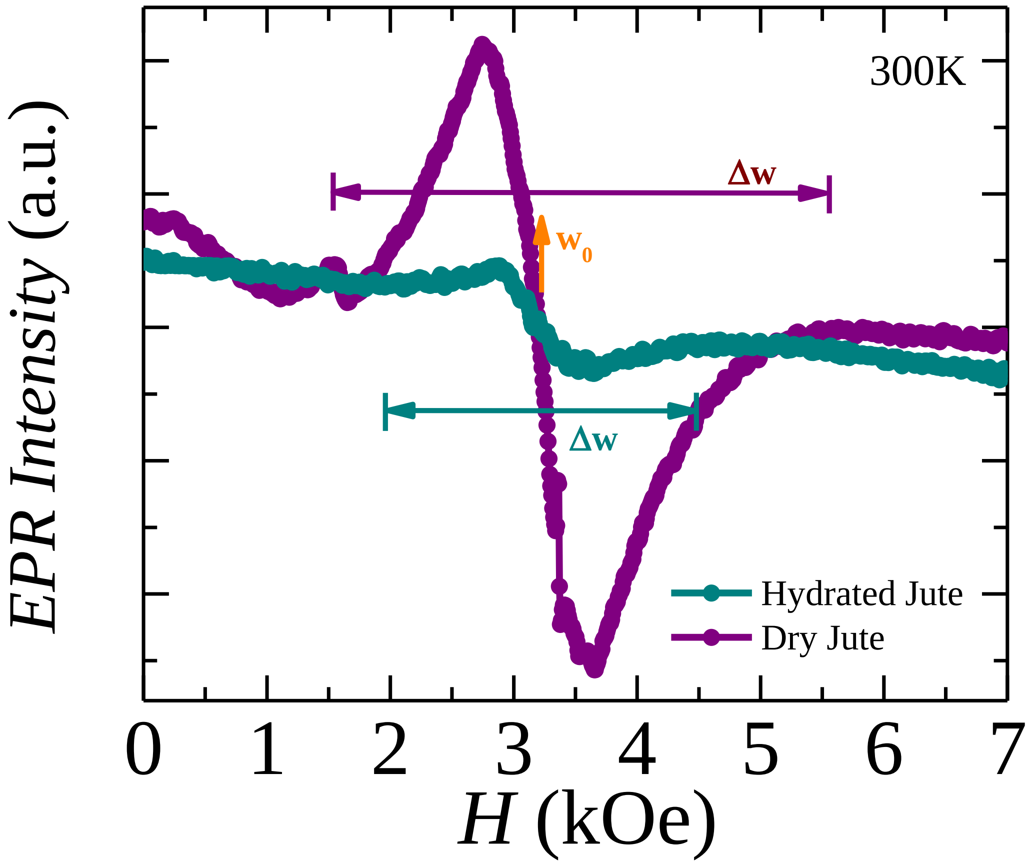

Extended Data Figure 5 | EPR study on jute sample. First derivative of Electron paramagnetic resonance (EPR) absorption spectra is shown as a function of applied magnetic field (*H*) for dry (purple) and hydrated (dark cyan) jute fiber. It shows the center of the EPR absorption spectrum (vertical arrow, w_0) does not shift, although there is decrease in the width (Δw) of the spectrum with hydration. It also shows shrink in the intensity of the signal when it is in hydrated state compared to dry state similar to SCM_{BMW} and human hair (Fig. 4 main manuscript).

Device made for dielectric constant measurement

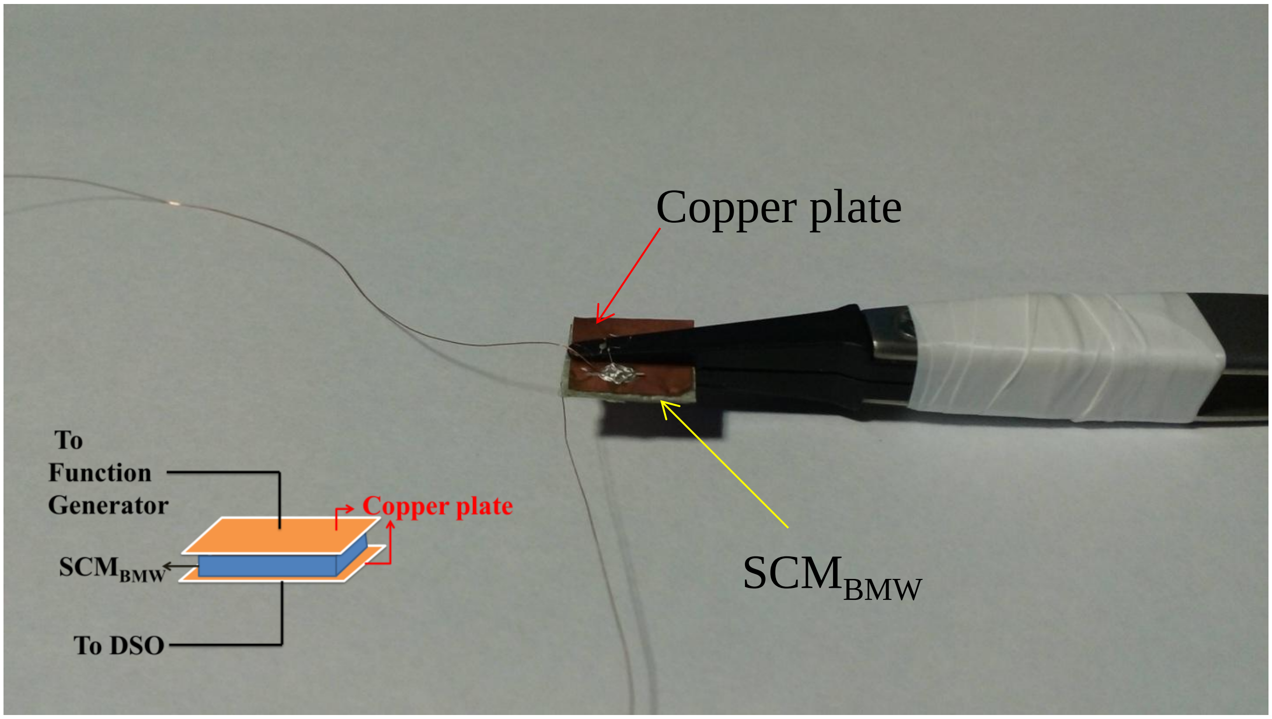

Extended Data Figure 6 | Dielectric measurement of hydrated SCM$_{BMW}$. Figure shows device made for measuring the dielectric constant (ε_r) of SCM$_{BMW}$. A small square piece of SCM$_{BMW}$ (length = 1 cm, width = 1 cm, thickness = 0.5 mm) is sandwiched between two thin copper pieces on which we made the contacts as shown in the figure. First we hydrated the SCM$_{BMW}$ and then used this device for the measurement. In the left corner we have shown the schematic representation of the experiment.

Frequency response of hydrated SCM_{BMW}

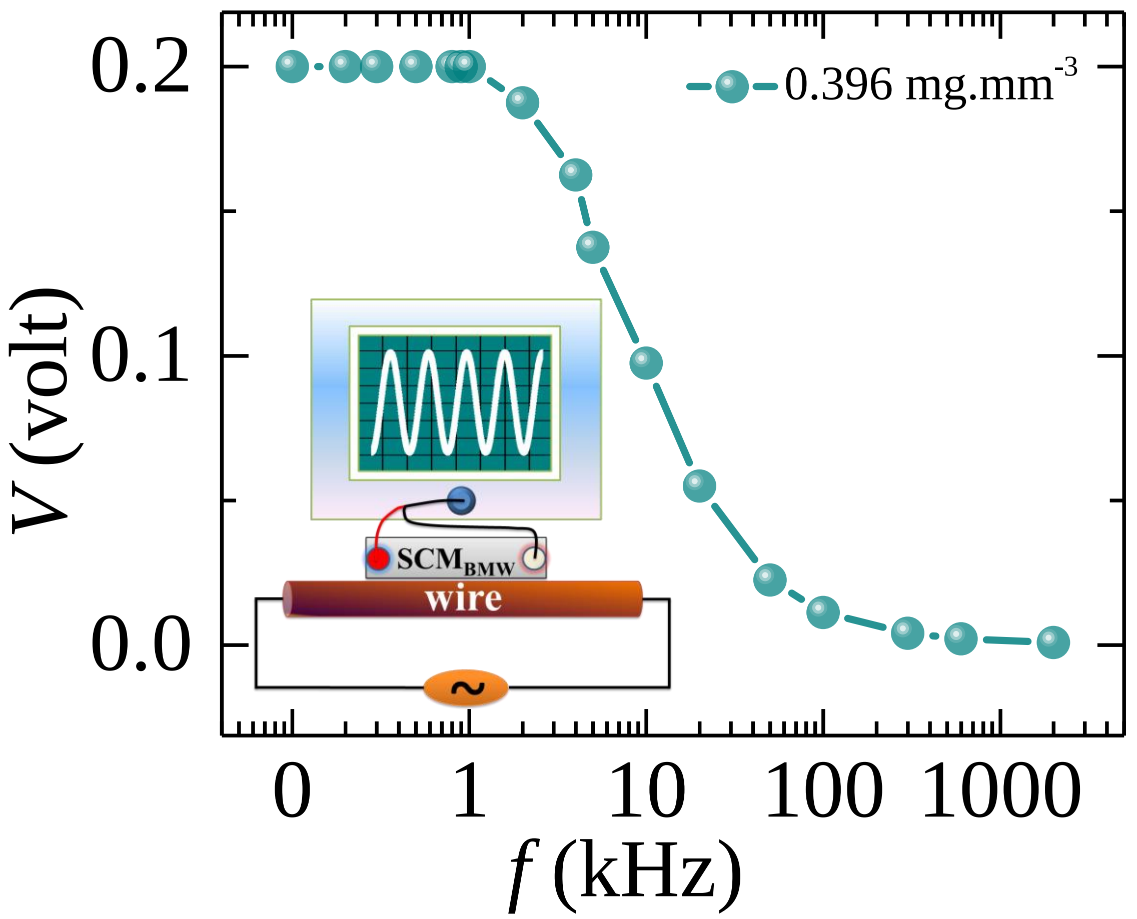

Extended Data Figure 7 | Frequency dependency of signal picked up by hydrated SCM_{BMW}. Figure shows frequency response of SCM_{BMW} at fixed water concentration (*d*). Hydrated SCM_{BMW} (*d* = 0.396 mg.mm^{-3}) is kept in direct contact of a wire through which we are sending signals of different frequencies and response of SCM_{BMW} is measured using Yokogawa DL9000 series digital storage oscilloscope (DSO). This shows that at higher frequencies (>1 kHz) the amplitude of the signal picked up by SCM_{BMW} falls rapidly i.e. it is more sensitive to pick up frequencies < 1 kHz. We already have shown in Fig. 7 of main manuscript that it is able to pick up low frequency signals (< 3 Hz). This shows that it has property of picking up signals within a range of frequencies (1 Hz – 1 KHz) and can be used as low pass filter.

Signal pick-up from human body by SCM_{BMW} at different water concentration

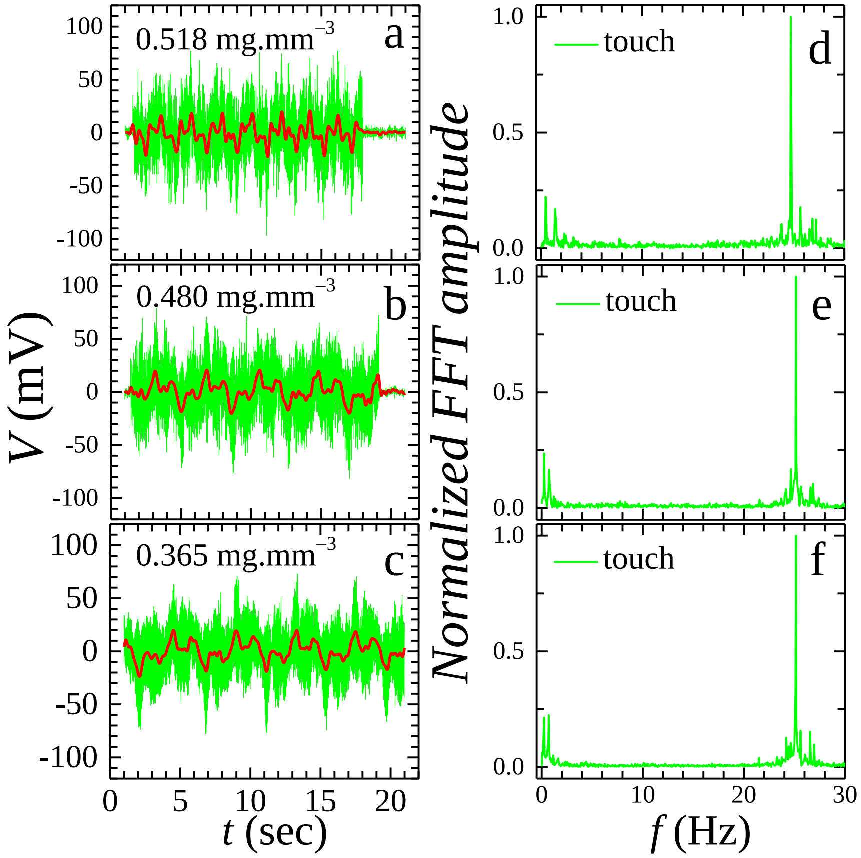

Extended Data Figure 8 | Low frequency signal picked up by hydrated SCM_{BMW}. Signals in Figs. **a-c** are obtained (a 49-51 Hz band block fast fourier transform (FFT) filter is applied on the original signal in order to remove signals coming from the nearby electrical wires carrying electrical currents at 50 Hz) at different hydration levels of the membrane in contact with human skin using Yokogawa DL9000 series DSO. The red curve in Fig a-c is obtained after a 3 Hz low pass FFT filter is applied on green curve data to extract only the low frequency modulations present in $V(t)$ (similar to Fig. 7 in the main manuscript). Figure **d-f** show the FFT spectrum of the corresponding green $V(t)$ curves in Figs. **a-c**. The FFT spectrum clearly shows peaks bunched near 22 to 28 Hz and peaks below 3 Hz (Supplementary Material Video 2). It appears these hydrated membranes are sensitive in picking up low frequency electric field modulation when in touch with human skin within a range of water concentration. At low water concentration we observe no signal picked up by SCM_{BMW}.

Supplementary Information, Video S1

Signal is picked up by hydrated SCM_{BMW} when it is near a wire carrying 50 Hz signal and as it move away from the wire, amplitude of the signal decreases, which corresponds to Fig. 6a in the main manuscript.

Supplementary Information, Video S2

Signal is picked up by hydrated SCM_{BMW} when it is in human contact. A movie of signal being picked up by hydrated SCM_{BMW} when it is in touch and when it is in air, which corresponds to Fig. 7.
